#  DETECTION OF NEW HIV CASES UNDER EPIDEMIC CONTROL - WHEN IS IT THE RIGHT TIME TO REVIEW HIV TESTING ALGORITHMS FOR COUNTRIES NEARING THE 95-95-95 MILESTONES? CASE STUDY -BOTSWANA.

**DOI:** 10.21010/Ajidv19i1.8

**Published:** 2024-10-25

**Authors:** NKOANE, Tapologo, QUTIESHAT, Abubaker

**Affiliations:** ^#^Graduate School of Business, University of Zambia, Lusaka, Zambia; ^*^ University of Dundee, Dundee, Scotland, UK

**Keywords:** HIV testing algorithm, HIV epidemic control, 2-test strategy, 3-test strategy, WHO 2019 HIV Testing Services

## Abstract

**Background:**

Accurate diagnosis of human immunodeficiency virus (HIV) infection is dependent on using established national HIV testing algorithm. The purpose of this study was to review published articles to identify, and apply lessons learned to determine factors affecting transition of HIV testing algorithm for countries that have attained HIV epidemic control.

**Materials and methods:**

We systematically searched peer-reviewed articles from online scientific databases; PubMed and Google Scholar from January 2019 to March 2024, using defined search phrases to extract articles. The defined search terms targeted articles focusing on issues of attained epidemic control and transitioning of HIV testing algorithm. Descriptive analysis was used to develop themes to answer the research question.

**Results:**

The findings show that countries should consider the following areas before changing their HIV testing algorithm: (i) evaluate the current national HIV testing algorithm to determine the Positive Predictive Value (PPV), which should be ≥ 99%, (ii) evaluate test kits bearing both CE and FDA certification and are WHO pre-qualified (iii) 4^th^ generation Rapid Diagnostic Tests (RDTs) are less sensitive than laboratory-based 4^th^ generation assays, (iv) pilot the proposed algorithm before country-wide roll-out, (v) country specific policies, and framework are required to guide transition, and (vi) a combination of 4^th^ and 3^rd^ generation RDTs can achieve desired PPV.

**Conclusion:**

Transitioning HIV testing algorithm for countries reaching HIV epidemic control is a multistep process which considers the PPV of the current algorithms, considers policies and framework to guide the process and, evaluates a combination of 4^th^ and 3^rd^ generation RDTs.

## Introduction

HIV became a public health problem about forty (40) years ago when the first case was recorded in 1981 in the United States of America (USA), (Robertson, 2005). Since then, HIV has been spreading globally with infections reaching 84.2 million in 2021, (UNAIDS, 2022). HIV remains a global health threat with an estimated 38.4 million people living with HIV (PLHIV) in 2021, only 85% of them knew their HIV status; roughly 5.9 million PLHIV did not know their status (UNAIDS, 2022). At the end of December 2022, an estimated 39 million people globally were living with HIV, 1.3 million people became newly infected with HIV, 630 000 people died from AIDS-related illnesses and 29.8 million (76%) of all people living with HIV were accessing antiretroviral therapy (UNAIDS, 2023).

Due to the global aggressive upscaling of HIV treatment, since 2001, an estimated 16.5 million AIDS-related deaths have been prevented. Countries are continuing to intensify efforts to eliminate HIV by 2030, with 40 countries on track to achieving the Joint United Nations Programme on HIV/AIDS (UNAIDS) target of 90% AIDS-related mortality reduction by 2030 (UNAIDS, 2021). Despite the success in the rollout of HIV treatment, only 86% of all people living with HIV knew their HIV status in 2022 (UNAIDS, 2023). Additionally, the UNAIDS global HIV statistics report of 2023 (UNAIDS, 2023), indicate that the highest burden of HIV new infection is concentrated in sub-Saharan Africa where in 2022, young women and girls accounted for 63% of new infections. Despite efforts to increase HIV testing, the rate of undiagnosed HIV infection among men and young people, including young women, and key populations remains way too high (UNAIDS, 2023). HIV diagnosis is essential to the success of the HIV response; it facilitates linkage of clients to the right intervention, like prevention strategies, counselling services or HIV treatment (WHO, 2021a, b). Accurate HIV diagnosis is central to bringing countries closer to achieving the target of ending AIDS as a public health threat and situating HIV treatment and care as part of universal healthcare coverage (Ford *et al.*, 2021)

According to Botswana’s most recent population-based survey- “The Fifth Botswana AIDS Impact Survey, BAIS V,” conducted in 2021, the disease burden was pivoting towards the adult (15-65 years) population as they presented a prevalence of 20.8%, and incidence of 0.2%, while prevalence among children (0-14 years) was 0.8% (National AIDS and Health Promotion Agency, 2023). Additionally, the survey highlighted population groups of males (15-24) and young people (15-24) as struggling to achieve the first 95 of the UNAIDS 95:95:95 targets at 89.1% and 84.5% respectively; hence the need to have innovative testing strategies to reach these populations.

However, it is encouraging that HIV high burden countries such as Botswana, Eswatini, Rwanda, Zimbabwe and the United Republic of Tanzania have attained the UNAIDS 95-95-95 targets, while 16 more countries, including eight (8) in sub-Saharan Africa are close to achieving these targets (UNAIDS, 2023). Whilst these achievements give hope for an AIDS-free society, what are the implications of this scenario in terms of national testing algorithms since majority of people living with HIV have been diagnosed and are on treatment? The prevalence of undiagnosed PLWH is under 5% in some countries mimicking a low prevalence setting. According to World Health Organization (WHO), between 2010 and 2018 in eastern and southern Africa, the number of adult PLHIV unaware of their HIV status decreased from 6.1 million to 2.8 million (WHO, 2019b). The proportion of adults with undiagnosed HIV decreased from 2.8% to 1.0% and in countries with 20% HIV prevalence and above, in 2018, national HTS positivity was close to, or below 5%. In 2019, in response to changes in the HIV epidemic, WHO encouraged countries to move towards using three consecutive reactive HIV rapid tests to provide an HIV-positive diagnosis (WHO, 2019a, b). Despite this recommendation and for Botswana to achieve the UNAIDS 95-95-95 targets, the country has not yet moved to three consecutive tests HIV diagnosis algorithm. Attainment of the UNAIDS 95:95:95 HIV epidemic control demands a review of the national HIV testing algorithm to ensure accurate HIV diagnosis.

### HIV elimination strategies over time

Strategies to mitigate against the spread of HIV are many and have evolved with the pandemic. Primary prevention strategy messages were pioneered in the early days of the pandemic and have been credited with containing the pandemic before antiretrovirals (ARVs) became widely available (Okware *et al.*, 2005). However, the contribution of the ABC (abstain, be faithful, use condoms) prevention strategy towards a healthier nation could not be quantified, especially *abstinence* since, culturally, in most developing countries, men dominate the sexual engagement decisions (Murphy *et al.*, 2006), hence women have no control over when and how to engage sexually. Additionally, evidence from a 15-year period generated from over twenty (20) PEPFAR (the U.S. President’s Emergency Plan for AIDS Relief) supported countries found no association between HIV reduction and two elements of the ABC (i.e., abstinence and faithfulness) strategy (Lo *et al.*, 2016). Other strategies that followed, including the elimination of HIV transmission from mother to child (EMTCT), safe voluntary medical male circumcision (VMMC), and biomedical interventions like pre-exposure prophylaxis (PREP) and post-exposure prophylaxis (PEP), have significantly reduced HIV infections (PEPFAR, 2021).

### HIV diagnosis, a gateway to HIV elimination

HIV testing is the gateway to treatment, the earlier that a person is diagnosed as living with HIV, the earlier they can start treatment. Effective treatment does not only afford infected people to live long and healthy lives, but it is also a great HIV prevention tool which averts onward HIV transmission. In 2016, WHO introduced the “test and treat strategy” (WHO, 2016), which ensures that persons who tested HIV positive are started on long-life antiretroviral medication immediately to preserve quality of life and lower the chances of viral transmission infection to more people. Furthermore, in 2014, UNAIDS announced audacious targets for the global response to HIV, named the 90-90-90 strategy - that 90% of people living with HIV (PLHIV) know their status, 90% of diagnosed PLHIV are on treatment and 90% of PLHIV on treatment achieve an undetectable viral load, by 2020 (UNAIDS, 2016). By the end of 2020, the UNAIDS targets were revised, and new targets were set as 95:95:95 by 2025, replacing the 90:90:90 targets. However, to ensure people are started on treatment, they must be found (located) and accurately diagnosed. With the evolving pandemic, strategies for locating the remaining infected people have advanced to include partner notification services (PNS) and HIV self-testing (Johnson *et al.*, 2019). Different testing strategies have helped in the fight against HIV by ensuring that each population group is reached.

### Available Diagnostic Test

Various tests to accurately diagnose HIV have been developed over the last 40 years of coexisting with HIV. Majority of testing worldwide is based on serology testing, either in the design of enzyme immunoassays (EIAs) or HIV rapid diagnostic tests (RDTs), and the chemistries of these tests have been improved over the years to enhance diagnosis accuracy; currently, there are five generations of these test kits (Parekh *et al.*, 2018). The test principles and chemistries define the test kit generation, hence there are different generations based on the time the kit was introduced. Each subsequent generation offers an improved ability to correctly identify an infection better than the predecessor test kit. The 1^st^ generation test is based on the detection of host (person’s) immunoglobulin G (IgG) antibodies in the blood or blood products (Parekh *et al.*, 2018), while the 2^nd^ and 3^rd^ generations are based on the detection of host antibodies IgG and immunoglobulin M (IgM); the 4^th^ and 5^th^ generations are based on the simultaneous detection and differentiation of HIV antibodies (IgM, IgG) and HIV antigen p24 (protein 24) narrowing infection detection to as early as two weeks after exposure (Parekh *et al.*, 2018; Bangalee *et al.*, 2021). Although RDTs have similar designs to EIAs, their advantage is in the simplified testing package requirements, offering the flexibility of the test to be performed by non-laboratory personnel in different settings, including the community, hence opening the possibilities of reaching and testing many people. While EAIs and RDTs should produce comparable results, the shorter incubation period of RDTs may result in missed infection detection (Parekh *et al.*, 2018). Furthermore, Nucleic Acid Test (NAT), available to detect infection earlier than serology-based tests (Merrick *et al.*, 2022), is restricted to the laboratory setting.

### Disease prevalence and effect on diagnosis

HIV testing dynamics change as more people are diagnosed and are started on treatment. This results in decreased total number of people who are HIV infected and remain undiagnosed in the population and this affects the predictability of identifying infected persons; the parameter known as positive predictive value (PPV). The accuracy of a test kit depends on its sensitivity i.e., the ability to correctly identify disease and the specificity i.e., the ability to correctly rule out the disease. Sensitivity and specificity are not enough when designing a testing algorithm. The prevalence of the disease must be taken into consideration as it affects the PPV. Both PPV and negative predictive (NPV) values are influenced by the prevalence of disease in the population that is being tested; in a setting of high disease prevalence, it is more likely that persons who test positive, truly have the disease than if the test is performed in a population with low prevalence (Molinaro, 2015). A combination of different test kits in the design of a test algorithm helps to improve the PPV. WHO recommends a PPV of at least 99% (WHO, 2019a, b; WHO, 2021b), that is, allowing only a 1% chance of reporting false positive results. The WHO guidance (WHO, 2019a, b; WHO, 2021b) therefore recommends a transition to 3-test serial testing regardless of the national HIV positivity rate, to guarantee a ≥99% PPV.

### Determining an HIV testing algorithm for different settings.

Continuous monitoring of the HIV pandemic at the population level is critical because it provides the PPV which is an indication of the accuracy of identifying infected people at a given timepoint. PEPFAR encourages and funds countries to conduct a population-based HIV impact assessment (PHIA) surveillance every three to five years to assess progress towards HIV elimination. National HIV prevalence is among the many outcomes of PHIA, and it is used as a guide in the design of the HIV testing algorithm. Over the years, WHO has been designing HIV testing algorithms for different HIV prevalence and encouraging countries to follow the algorithm that suits their HIV pandemic. The WHO testing guideline started in 2015, advising countries with high prevalence (>5% positivity rate) to transition from a parallel to a 2-test-serial RDT algorithm (WHO, 2015). The implementation of the test-and-treat strategy further reduced the remaining population of HIV undiagnosed individuals as more people were started on HIV antiretroviral treatment. The PHIAs, conducted after the intervention of the test-and-treat strategy, found that most countries were reporting less than 5% of HIV prevalence among the undiagnosed, hence the need to transition from a 2-test to a 3-test HIV diagnostic algorithm to avoid misclassifications (Patel *et al.*, 2022).

Since the introduction of the “test and treat” strategy, HIV diagnosis has remained the sole qualifier for HIV treatment. HIV misclassification is a concern as it can put an HIV-free person on lifelong medication and cause physiological, social, and financial burdens. HIV misclassification has been described as resulting from a lack of following the established testing algorithm (Skovdal *et al.*, 2022; Dupwa *et al.*, 2019); using a suboptimal testing algorithm (Patel *et al.*, 2022); or the wrong choice of test kits (Adetunji *et al.*, 2019). Adopting the WHO recommendation of 3-serial testing to diagnose HIV needs to be approached with supporting evidence. There is concern, especially from resource-limited, disease-burdened countries like South Africa, that transitioning to the 3-test strategy will overwhelm the already resource-constrained health system, increase results turnaround time, and increase the complexity of results interpretation (Mashishi *et al.*, 2021). Furthermore, contrary to current beliefs, evidence from Swaziland (now Eswatini), Malawi, and South Africa shows that the currently available 4^th^ generation (combination of antibody and antigen; HIV-1 Ag/Ab) HIV RDTs kits are not ideal for detecting new infections in Sub-Saharan Africa (Adetunji *et al.*, 2019). However, evidence from a recent study of a new version of Determine HIV-1/2 Ag/Ab 4^th^ RDT shows promising results of early detection of HIV (Wratil *et al.*, 2020). As more countries reach HIV epidemic control and are forced to follow transition guidelines, there is a need to provide guidance on the approach to transition correctly. The purpose of this study was to review the peer-reviewed published articles focusing on HIV epidemic control attainment and HIV testing algorithm transition to identify, emphasize, and apply lessons learned to provide guidance to smoothen the transition of the HIV testing algorithm when epidemic control is attained.

## Materials and Methods

The study evaluated peer-reviewed literature focusing on HIV testing algorithms and HIV epidemic control. The methodology used was analysis and evaluation of published research to answer the question, “*What can the published literature highlight as lessons learned and determinates for HIV algorithm transition as countries reach HIV epidemic contro*l?”.

Boolean operators such as “AND”, “NOT”, “OR” were used to extract articles from the online journal databases namely, PubMed and Google Scholar using different combinations of search words as described by (Vogus *et al.*, 2015; Joulaei *et al.*, 2018; Ali *et al.*, 2019; Fonner *et al.*, 2020). The search was done by combining each word from the column “HIV testing strategies search terms” with each word from the column “Health area search terms” (Table 1). The search was done by one person. Table 2 depicts the process that was followed to identify the article that contributed to this study and the findings are summarised in Table 3.

**Table 1:** Key search for online journal databases using Boolean logic.

HIV testing strategies search terms (using “OR”)	Combination of each search words from the two columns	Health area search terms (Using “OR”)
3 test strategy	AND	HIV Epidemic control
2-test strategy		WHO 2019 HTS guidelines
HIV testing algorithm		Low HIV prevalence
		HIV misclassification

**Table 2:** Summary of selection process of articles extracted from PubMed and Google Scholar

Article inclusion process	Total
Total articles retrieved.	763
Excluded based on relevance of title and abstract, not a full article, not original article (i.e., reviews, commentary, letters to editor)	32
Exclusion after reading full text (not contributing to reach question)	13
Articles analysed	14

Application of inclusive and exclusive criteria was applied on the 673 retrieved articles, only 14 articles eligible for analysis.

**Table 3:** Systematic review of factors to consider prior to HIV algorithm transitioning

Author, title	Year of study publication	Study geographical area	Study design	Study objective	Characteristics	Major conclusion and contribution to this study
1.Patel *et al.,* Performance of HIV rapid testing algorithm in Nigeria: Findings from a household-based Nigeria HIV/AIDS Indicator and Impact Survey (NAIIS).	2022	Nigeria	Nation Population based HIV household survey	Analysis of performance of HIV testing algorithm The evaluated HIV algorithm design: serial testing comprising of (T1) Determine HIV- 1 /2, Unigold HIV- 1 /2, (T2) and StaPak HIV- 1 /2, (tie breaker)	Conducted in 2018 Participants_ 18 moths to 64 years 204, 930 samples Rapid HIV field test confirmed by lab-based HIV tests (Geenius, Western blot)	Important to evaluate algorithm to decide when to switch to new one. National algorithm PPV of 94.5%. WHO recommended ≥99% PPV.
2.Petersen *et al.,* Reducing False-Positive Results with Fourth-Generation HIV Testing at a Veterans Affairs Medical Left,	2021	USA	Case study Retrospective study	Impact on the false-positive screening rate when switching from 3^rd^ to 4^th^ HIV generation for veteran patient population	Reviewed test reports from March 2016-Feb 2017; 3^rd^ Gen; n= 7516 and March 2017-Feb 2028: 4^th^ Gen, n=7802 Veteran population Participants aged 20 years and older. Lab based assays.	Lab based 4^th^ gen can be used to reduce the number of false positive. 4^th^ gen more specific than 3^rd^ gen assay. Increased efficiency and cost savings
3.Augusto *et al.,* High level of HIV false positives using EIA- based algorithm in survey: Importance of confirmatory testing	2020	Mozambique	Cross sectional HIV Household survey	The impact of using Bio-Rad Geenius™ HIV-1/2 Murex HIV-1/2) used as supplementary assay as a confirmatory assay to HIV results tested by National HIV algorithm 2-serial Enzyme Immunoassay (EIA), Vironostika-HIV-1/2 and Murex HIV-1/2)	households in 2015 Ab based assays general population. 11690 specimens	A more specific confirmatory testing should be added to the EIA-based algorithms to reduce false positive results. 31.5% false positive with EIA algorithm, overestimating prevalence
4.Martin *et al.,* Rapid Testing Algorithm Performance in a Low-Prevalence Environment	2020	USA	Multi sites	Performance of an HIV screening algorithm where T1 HIV-1/2 Ag/Ab Combo test (T1) with verification of Ab by a second different second, rapid test (T2). To determine utility of antigen biomarker	310,785 rapid tests using 4^th^ gen RDT and confirmed by 3^rd^ gen Rapid test assay. 3-year study (2015-2018). Low HIV prevalence setting	The sensitivity of the RTA, where T1 is 4^th^ gen and T2 is 3^rd^ Gen was 99.36%, and specificity was 98.38%. Antigen biomarker increases sensitivity.
5.Huang *et al.,* Evaluation of a two-test strategy for HIV screening in a low-prevalence setting and the indications for optimizing clinical management	2023	China	retrospective analysis	To evaluate the feasibility of utilizing the two-test strategy to optimize clinical management for initial HIV screening-positive patient. In a hospital.	Analysis of HIV data from 2017–2020 220,558 participants (inpatients & outpatients) 2-test algorithm (lab-based): 1^st^ Architect HIV Ag/Ab (Combo Assay test) 4^th^ Gen and second test (Abon Biopharm) a 3^rd^ Gen assay. Western blot as confirmatory	Use of 4^th^ generation alone gives high false positive rates in low HIV prevalence. HIV screening using 4^th^ Gen assay, followed by a 3^rd^ Gen assay gives sensitivity of 100% and a desired PPV of 98.7%
6. Yoo, S. J. Clinical Utility of HIV-1/2 Ab Immunochromatographic Assay as an Additive Test for HIV Screen-Positive Patients	2020	South Korea	Retrospective	Utility of Rapid Antibody diagnostic tests in settings where 4th gen assays are used	HIV Antibody screen (July 2011-May 2019).158,431 specimens. Lab based 4th Gen assay. Rapid Test – 3rd Gen	An algorithm comprising of both 3rd and 4th generation assays helps to prevent false positives reported by 4th gen and false negatives by 3rd Gen assays hence facilitating in early decision making for patient management
7.Sirivichayakul *et al.,* Ability of Alere™ HIV Combo to diagnose acute HIV infection is based mainly on HIV-1 p24 antigen detection.	2021	Thailand	Retrospective Used stored plasma	Point of care 4^th^ generation antigen/antibody HIV assay	50 HIV acute HIV samples collected between January 2014 to March 2015 Lab-based 4^th^ generation acute HIV samples retested with Determine HIV-1/2 Ag/Ab Combo test	4^th^ generation rapid test, though less sensitive than machined-based 4^th^ gen RDT assay is a good replacement of lab-based 4^th^ generation assay in high prevalence settings since could detected 75% of acute individuals missed by 3^rd^generation antibody assay.
8.Mashishi *et al.,* The evolving HIV epidemic and its impact on the HIV testing algorithm: Is it time to change the HIV testing algorithm in South Africa*	2021	South Africa	Case study	Overview of the HIV epidemic and impact on HIV testing	Country context review to guide policy	Concerns that 3-test strategy will increase test volume hence cost, turnaround time and general resources.3-test may pose results interpretation challenges. There is need to have standardized interpretation criteria.
9.Barr et al., Treatment-adjusted prevalence to assess HIV testing programmes	2021	Sub-Saharan Africa	Retrospective	Utilization of the treatment-adjusted prevalence indicator to calculate the country’s HIV positivity rate (considering both ART coverage and national HIV prevalence) in Kenya, Malawi, Zimbabwe, South-Sudan to guide policy.	National program to mathematically calculate true HIV prevalence among the treatment naïve adult population.	Utility of treatment- adjustment indicator to guide change of HIV algorithm. Practical application to select the appropriate, cost-effective HIV testing algorithm. Helps in projecting treatment costs. Helps to analyze the effectiveness of targeted HIV testing.
10. Manjate et al., Laboratory-based evaluation of the 4th-generation Alere HIV Combo rapid point-of-care test	2024	Mozambique	Retrospective	Evaluation of the 4th Gen RDT Alare Combo to detect acute and chronic HIV infection among sexually active women in 3 health lefts in Maputo.	920 field tested samples between February 2018 and January 219. Confirmed by 3rd generation RDT and lab-based Ab/Ag and molecular tests. Reference panels were also used.	4th generation Alere RDT HIV combo’s antibody component has high sensitivity and specificity
11. Qiu et al., An improved HIV antigen/antibody prototype assay for earlier detection of acute HIV infection	2021	USA	Prospective and stored samples	Evaluate performance of a newly developed assays against the FDA approved assays for detection of early HIV infection.	Kit evaluated against five FDA approved kits with variety of samples including standard and reference panels, samples from individuals with low-risk HIV infection, blood donors	Non-FDA approved Kit had acceptable sensitivity and specificity as described by WHO. Thorough evaluation of test kit, using the available standards and different HIV strains critical prior acceptance of kit for population testing
12.Krasowski et al., Real-World Clinical Performance Evaluation of a Fourth -Generation HIV Antigen/Antibody Differentiation Testing	2021	USA	Performance evaluation	Assessment of Elecys HIV Duo assay against gold standard algorithm from US and Non-Us citizens.	Labs based testing of 4th Elecys assay against Architect using a HIB generically diverse samples to detect antigen and antibodies concurrently and report them individually/separately, 10121 specimens from different populations.	laboratory HIV Ag/Ab immunoassay 4th gen assay gave 100% sensitivity (true pos); ensures non-miss of all persons infected with HIV regardless of genetic make-up of the HIV
13. Demir et al., Evaluation of the diagnostic performance and optimal cutoff value of a fourth-generation ELISA, VIDAS HIV-1/2 Duo Ultra assay, in a low-prevalence country	2020	Turkey	Retrospective	Use of VIDA HIV Duo Ultra assay to identify both early and established HIV infection	11642 ELISA positive samples confirmed with VIDAS HIV Duo Ultra test. Low prevalence setting	VIDA HIV Duo Ultra assay as a confirmation to a reactive ELISA assay can be used to correctly identify both early and established HIV infection. Evaluation of emerging tests vital to improve algorithm. Algorithms containing lab-based 4th Gen assay is cost effective and can identify all true positives, even in low prevalence setting.
14.Fajardo et al., Country adoption of WHO 2019 guidance on HIV testing strategies and algorithms: a policy review across the WHO African region	2023	Multi Country	Documents analysis	Review of the HTS National Policies of WHO African region – 47 countries against the 2019 WHO Recommendation for HTS being: 1) use of serial testing, (2) use of a three- test strategy, (3) discontinuation of a tiebreaker (4) discontinuation of Western blot (5) Verification testing and (6) use of dual HIV/syphilis RDT in ANC	Country HTS policies Supporting documents Descriptive analysis	Slow adaption of recommendations, especially three test strategy. In Southern-African, only 1 country in had a policy but no implementation

**Abbreviation**: Ab, antibody; Ag, Antigen ANC, Antenatal Clinic; FDA, U.S. Food and Drug Administration; Gen, Generation; HIV, Human Immunodeficiency Virus; HTS, HIV Testing Services: Lab, Laboratory; RDT, Rapid Diagnostic Tests; T1, Test 1; T2, Test 2; WHO, World Health Organization; USA, United States of America; USA, United States of America

The search was done using specific, pre-defined search words in different combinations with the Boolean operators to search for articles from the online journal data bases to extract desired articles to answer the research question *“What can the published literature highlight as lessons learned and determinates for HIV algorithm transition as countries reach HIV epidemic contro*l?”.

### Eligibility criteria

Inclusion criteria were articles; (i) published after 2019, (ii) published in English, (iii) that were original (iv) and with full version available. Exclusion criteria included articles; published prior to 2019, where full version was not available, inappropriate data, inappropriate titles, reviews, editorials, and commentary.

### Data extraction

The final selection of articles followed the following steps: scanning the titles and abstracts of articles for relevance, reading the full text to gain context, and dropping those articles whose content did not address the research question.

The selected articles were read and analysed at least twice on separate days by one person and the data from the two time points were compared. Where there were discrepancies, a third final reading and analysis was done, and inclusion was based on the agreement of two analyses from different time points. If there was no concordance in any of the three readings, the article was excluded from the final analysis.

### Data Analysis

Data were synthesized from the extracted articles and themes were developed and categorized into distinct groups to address the research question, “*What can the published literature highlight as lessons learned and determinates for HIV algorithm transition as countries reach HIV epidemic control*?”. Table 2 depicts the process that was followed to identify the articles that contributed to this study. The initial search yielded 763 articles, but after applying inclusion and exclusion criteria, only 59 remained. A further 32 articles were excluded as they were either reviews, letters to the editor, or documentaries; 13 were rejected because they did not contribute to the research question. Only 14 articles were eligible to be analysed. Descriptive thematic analysis was used for data analysis (Table 3).

## Results

The following fourteen peer-reviewed articles, (Patel *et al.*, 2022; Petersen *et al.*, 2021; Augusto *et al.*, 2020; Martin *et al.*, 2020; Huang *et al.*, 2023; Yoo, 2020; Demir *et al.*, 2020*;* Sirivichayakul *et al.*, 2021*;* Manjate *et al.*, 2024 Mashishi *et al.*, 2021*;* Barr *et al.*, 2021*;* Qiu *et al.*, 2021*;* Krasowski *et al.*, 2021*;* Fajardo *et al.*, 2023), as indicated in Table 3, were included in this study. These articles cover factors that should be considered prior to transitioning from the current 2-test serial algorithm to 3-test serial algorithm for countries that have reached HIV epidemic control according to UNAIDS 95:95:95 targets.

The findings pointed to six considerations for countries to make prior to transitioning HIV testing guidelines, and these are: (i) the need to evaluate the current HIV testing algorithm to determine the Positive Predictive Value (PPV) which ideally should be above 99%. This PPV can be derived from Population-based HIV Impact Assessments (PHIAs) being carried out by countries (Patel *et al.*, 2022) or by employing Treatment Adjustment Indicator (Barr *et al.*, 2021), (ii) countries need to propose a new algorithm based on three tests and carry out pilot of the proposed algorithm prior to country-wide roll-out, in order to finetune the implementation and allowing updating the implementation protocols prior to country-wide transition, (iii) countries need to evaluate kits available in the market which includes those which are WHO-prequalified kits, CE and FDA certified to allow maximization of selecting the best test kits as only through evaluation can the true performance of test kit be established, (iv) countries should strive for a combination of 3^rd^ and 4^th^ generation RDTs in order to achieve the desired PPV of equal or greater than 99%, (v) countries need to be aware that fourth (4^th^) generation RDTs are less sensitive than laboratory-based 4^th^ generation assays, and (vi) countries should develop specific policies and frameworks to guide transition and implementation.

## Discussions

Our review shows that there is limited literature on this area as only a few countries are starting to reach epidemic control, and the majority have not switched to the 3-test strategy. Currently, Botswana, Eswatini, Rwanda, and Tanzania have met the 95-95-95 targets, and an additional eight countries in sub-Saharan Africa are close to achieving these targets (UNAIDS, 2023). As more countries reach epidemic control and transition their testing strategies, including testing algorithms, published data will become available, and there will be global representative literature to guide the transition and the choice of test kits by region, considering the predominant HIV strains in those regions. High burden countries such as South Africa with a population of 62 million (Statistics South Africa, 2024) and HIV prevalence of 12.7% (HSRC, 2023) are already grappling with issues relating to transitioning of their HIV testing algorithm to a three-test serial algorithm (Mashishi *et al.*, 2021). The South African main concerns with the three-test algorithm are increased testing volumes which will with no doubt increase the costs, and they are also concerned about turnaround times for returning results, presenting a logistical nightmare. A modelling study by Eaton *et al*. (2021) comparing a 2-test serial algorithm (WHO, 2015) to a 3-test serial algorithms (WHO, 2019a) across different pandemics (different positivity rates), showed that the 3-test algorithm maintained the desired PPV of >99% regardless of the HIV positivity rate, whereas the 2 -test performed poorly (< 99%PPV) when HIV positivity was less than 5%. This study showed that as countries move towards an epidemic control, there is need to transition to 3-test algorithm to improve finding new or undiagnosed persons. Eaton and colleagues admitted to a small cost incurred when implementing a 3-test algorithm due to the inherent addition of a 3^rd^ test to cater for the small volumes they deem a small trade-off to the risk of misclassification and enrolling an HIV naïve person into a long-life HIV treatment which may be observed when implementing a wrong algorithm for the country’s HIV prevalence.

More research is needed in choosing HIV test kits rather than solely relying on literature. Studies from the United States of America have shown that switching from 3^rd^ to 4^th^ generation RDTs reduced HIV misclassifications (Parker *et al.*, 2019; Petersen *et al.*, 2021). Similar studies in HIV high burdened countries in Sub-Saharan African showed no benefit in switching to 4^th^ generation RDTs (Duong *et al.*, 2014; Adetunji *et al.*, 2019; Manjate *et al.*, 2024). This contrasting evidence in the performance of 4^th^ generation RDTs needs to be understood. One explanation is that the kits are manufactured and optimized in a region where HIV subtype B is predominant, a strain different from the one circulating in Southern Africa, subtype C (Adetunji *et al.*, 2019).

Botswana national HIV testing program follows stringent quality control and assurance (QA/QC) with full participation in external and internal quality control testing (Ministry of Health-Republic of Botswana, 2016). The current Botswana 2-test HIV testing algorithm needs to be urgently evaluated to determine its PPV. This can be done using data from the fifth Botswana AIDS Impact Survey (Patel *et al.*, 2022), or the program data using treatment-adjustment prevalence indicator (Barr *et al.*, 2021). Nigeria recently evaluated its HIV testing algorithm using 2018 PHIA results (Patel *et al.*, 2022). The study showed a low PPV of 94.6% of 2-test HIV testing algorithm with the HIV prevalence of 1.4%. The implication of this study is that the 2-test HIV testing algorithm misses an estimated 5.5% in identifying HIV-infected individuals (Patel *et al.*, 2022). This study shows suboptimal performance of the current Nigeria 2-test HIV algorithm. This provided an impetus for Nigeria to change its HIV algorithm from 2-test to 3-tests.

Contrary to the general believe that due to the rigorous approval process by the FDA, FDA-approved kits perform better than CE-marked test kits (Mishra, 2017), field evidence using a large sample from three African countries (Livant *et al.*, 2017) found that the CE-marked kit accurately identified more diseased individuals than the same kit (same name, same manufacturer) bearing FDA-approval. Therefore, African countries should not rely only on the theoretical guidance but should perform kit validation for both CE-marked and FDA-approved test kits during the process of kit selection for algorithm design.

An algorithm that can correctly and effectively diagnose an infected person as early as possible helps to initiate treatment to suppress the viral replication, hence curbing the spread of the virus (Wang *et al.*, 2021). On a quest to find the few positives, highly sensitive assays such as 4^th^ generation RDT should be used. However, high sensitivity should not be the only determining factor when choosing tests for an algorithm. Leaning only towards more sensitive RDTs can result in misclassification of HIV positivity (Augusto *et al.*, 2020), leading to the initiation of the wrong persons into lifelong HIV treatment. Notably, there are few WHO pre-qualified 4^th^ generation RDTs (WHO, 2024). WHO verification protocol guides that the algorithm verification design should include at least four (4) RDTs from different brands and manufacturers as test 1 and the same for test 2 and 3, (WHO, 2021a) a guidance not achievable should a 4^th^ generation RDTs be a preferred first test. Therefore, caution should be exercised when selecting a combination of test kits to build the HIV testing algorithm. WHO recommends three consecutive serial-testing where the first test is highly sensitive (≥ 99% sensitivity) - to identify all HIV positives, followed by two highly specific (≥ 98% specificity) assays, tested serially - correct identification of HIV negatives to avoid misclassification (WHO, 2019a). The recommended WHO three-consecutive RDTs assay algorithm assures an attainment of the recommended 99% PPV threshold.

### Limitations

The limitation of this study is the small sample size of papers dealing with the subject; similar studies may need to be conducted in future as more literature becomes available. Additionally, there was less data available from sub-Saharan Africa as most of the reviewed articles were from more developed countries with low HIV prevalence. The review may have benefited from increased number of subject matter experts. Having two or three subject matter experts as authors usually helps to build a solid consensus and address discrepancies (Vogus *et al.*, 2016; Ali *et al.*, 2019).

## Conclusion

An accurate HIV diagnosis is essential, and care should be taken not to initiate a disease-free person into a life-long HIV antiretroviral drug. HIV testing algorithms should be designed to ensure PPV greater than 99% to have assurance of correct diagnosis. Transitioning test algorithms need to be guided by evidence derived from the national HTS program and national documents, population-based studies, and other country data. This study was able to identify the following critical factors to consider prior to transitioning the test algorithm, namely (i) evaluation of the current HIV testing algorithm to determine the PPV, (ii) evaluation of more rapid test kits preferably with both CE-marked and FDA approval certification, and WHO pre-qualification (iii) combinations of 3^rd^ and 4^th^ generation RDTs can achieve the desired PPV of equal or greater than 99% regardless of the HIV national positivity rate, (iv) there is need to implement a new algorithm in a step-wise fashion to manage complexity (v) it is important to note that 4^th^ generation RDTs are less sensitive than laboratory-based 4^th^ generation assays, however they are still more sensitive than 3^rd^ generation RDTs (Masciotra *et al.*, 2013, Wratil *et al.*, 2020), but 4^th^ generation RDTs pose a possible delay in establishing diagnosis and further cost as an antigen reactive/antibody non-reactive test requires either further testing using molecular or asking the client to come back for retesting after a couple of weeks when antibodies are detectable and (vi) there is need to develop country specific policies and frameworks, which should include, among other things, standardized results interpretation framework and costing of the algorithm.

In general, the findings indicate that a combination of highly specific and highly sensitive assays need to be included in the standard HIV algorithm to ensure accurate HIV diagnosis. Additionally, the country’s HIV algorithm needs to be assessed periodically to ensure that a desirable PPV threshold of ≥ 99% is maintained. Furthermore, the evaluation of test kits followed by a field pilot study is needed to assess the functionality of the proposed new algorithm prior to nation-wide implementation.

### Conflict of interest

The authors declare that there are no personal or financial relationships that may have inappropriately influenced them in writing this article.

**List of Abbreviations Used:**
Ab: Antibody;
Ag: Antigen;
EIA: Enzyme Immunoassays;
ARVs: antiretrovirals;
EMTCT: elimination of mother to child transmission ;
HIV: Human Immunodeficiency virus;
HTS: HIV Testing Services;
IgG: Immunoglobulin G;
IgM: Immunoglobulin M;
NAHPA: National AIDS and Health Promotion Agency;
NAT: Nucleic Acid Test;
NPV: Negative Predictive Value;
PHIA: Population-based HIV Impact Assessment;
PLHIV: People Living with HIV;
PEP: Post-Exposure Prophylaxis;
PEPFAR: The United States President’s Emergency Plan for AIDS Relief;
PPV: Positive Predictive value;
PREP: Pre-Exposure Prophylaxis;
RDT: Rapid Diagnostic Tests
UNAIDS: the Joint United Nations Programme on HIV/AIDS;
VMMC: Safe Voluntary Medical Male Circumcision;
WHO: World Health Organization.
